# Effect of antiretroviral therapy initiation time and baseline CD4^+^ cell counts on AIDS-related mortality among former plasma donors in China: a 21-year retrospective cohort study

**DOI:** 10.1080/16549716.2021.1963527

**Published:** 2021-10-01

**Authors:** Yongli Yang, Yang Li, Xuening Zhang, Weiping Zhang, Yanmin Ma, Xiaocan Jia, Theodore Gondwe, Yuping Wang, Xuezhong Shi

**Affiliations:** a Department of Epidemiology and Biostatistics, College of Public Health, Zhengzhou University, Zhengzhou, China; b Institute of STD/AIDS Prevention and Control, Henan Provincial Center for Diseases Prevention and Control, Zhengzhou, China; c Zhengzhou University Library, Zhengzhou University, Zhengzhou, China

**Keywords:** HIV/AIDS, antiretroviral therapy, treatment initiation, CD4^+^ cell counts, competing risk model

## Abstract

**Background:**

The conventional survival analysis model on HIV/AIDS prognosis is the Cox proportional hazard model, which deals with only one event type, death, regardless of the cause. Few studies have used a competing risk model to evaluate the predictors of AIDS-related mortality.

**Objective:**

To estimate the influence of antiretroviral therapy (ART) initiation time and baseline CD4^+^ cell counts on acquired immunodeficiency syndrome (AIDS)-related death among former plasma donors.

**Methods:**

A retrospective cohort study was conducted involving 11,905 human immunodeficiency virus (HIV) or AIDS patients in a high-risk area of Henan province in China between 1995 and 2016. Demographic and clinical data were collected. Sub-distribution hazard ratios (*sHRs*) for AIDS-related mortality with baseline CD4^+^ cell counts and ART initiation time were determined using a competing risk model.

**Results:**

Patients who initiated ART within 90 days of HIV/AIDS diagnosis (*sHR*: 0.24, 95% *CI*: 0.22–0.27) or had baseline CD4^+^ counts of >500 cells/μL (*sHR*: 0.23, 95% *CI*: 0.19–0.28) were associated with lower AIDS-related mortality risk. Patients with ART initiation time >1 year but CD4^+^ counts >350 cells/μL (*sHR*: 4.42, 95% *CI*: 3.30–5.91) had a higher AIDS-related mortality risk than those with ART initiation time >90 days but CD4^+^ counts ≤350 cells/μL (*sHR*: 4.33, 95% *CI*: 3.58–5.23).

**Conclusions:**

Our results demonstrate that patients with high CD4^+^ cell counts and late ART had a 9% higher risk of AIDS-related death than those with low CD4^+^ cell counts and early ART. This study confirms the great significance of immediate ART initiation among former plasma donor HIV patients in China.

## Background

Human immunodeficiency virus and acquired immunodeficiency syndrome (HIV/AIDS) continue to be major global public health issues. According to the Chinese Center for Disease Control and Prevention statistics, there were 820,756 individuals living with HIV/AIDS in China in 2018, and approximately 253,031 individuals who had died from AIDS-related causes. A major factor in the widespread incidence of AIDS in China is that thousands of small, illegal, commercial plasma collection centers were established in rural areas of China between 1990 and 1994 [1]. The re-use of syringes and the subsequent re-infusion of mixed red blood cells led to most former plasma donors (FPD) being infected with HIV through blood transmission in China, particularly in Henan province. Henan province continues to be one of the worst affected Chinese provinces in terms of HIV/AIDS [2].

Previous studies found that immediate antiretroviral therapy (ART) when CD4^+^ counts were >500 cells/μL could reduce mortality [3]. However, patients infected via blood transmission had higher plasma HIV loads and lower CD4^+^ cell counts than patients infected through other modes of transmission [4]. When the World Health Organization (WHO) released the second edition of their consolidated guidelines on the use of antiretroviral drugs for treating and preventing HIV infection, it recommended lifelong ART for all HIV/AIDS patients regardless of clinical status or CD4^+^ cell counts to reduce AIDS-related mortality [5]. We therefore sought to explore the impact of ART initiation time on AIDS-related mortality in patients with high and low CD4^+^ cell counts.

HIV/AIDS patients may die of non-AIDS-related diseases. The occurrence of death from non-AIDS-related diseases necessarily prevents the occurrence of death from AIDS related diseases; these causes of death compete with each other [6,7]. Conventional survival analysis studies considered death from non-AIDS-related causes as censored data rather than competing events, which overestimated the cumulative incidence function [8] and led to bias in the effects of covariates [9]. A competitive risk model can solve this problem, despite it being rarely used in the studies of HIV/AIDS prognosis and its performance rarely evaluated [10].

The primary aim of the present study was to explore the effect of ART initiation time, CD4^+^ cell counts, and other prognostic factors on AIDS-related death among a cohort of FPD using a competing risk model. We also sought to evaluate the performance of this competing risk model.

## Methods

### Study population and data collection

This study is a retrospective cohort involving 13,579 HIV/AIDS patients from Zhumadian city in Henan Province between 1995 and 2016. Data were from National HIV/AIDS Integrated Information Management System. All patients were confirmed by enzyme-linked immunosorbent assay (ELISA) and Western blot analysis. HIV-infected patients and AIDS patients were followed up by trained interviewers every six or every three months, respectively. Demographic information (gender, age at diagnosis, educational level, marital status, and occupation) and clinical characteristics (mode of transmission, disease stage at diagnosis, CD4^+^ cell counts, and time of ART initiation) were collected. In accordance with the time from HIV diagnosis to ART initiation, the whole population was classified into the immediate ART group (initiating treatment within 90 days of diagnosis), the delayed ART group (initiating after 90 days of diagnosis but no longer than one year after), and the late ART group (initiating after one year of diagnosis).

Only patients aged over 15 were included in this study because diagnostic criteria and severity of disease differed from patients below 15 years of age. Non-native patients (n = 35), patients without plasma donation information (n = 572), patients without follow-up information (n = 264), patients without ART information (n = 729), and cases of logical error (n = 74) were excluded. The present study was finally limited to 11,905 HIV/AIDS patients who met the eligibility criteria. This study was approved by the Ethics Committee of the Henan Center for Disease Control and Prevention (2019-KY-005-02).

### Definition of outcome

We defined AIDS-related death as the event of interest and non-AIDS-related death as the competing event. Causes of death were specified based on the International Classification of Diseases 10th revision (ICD-10). AIDS-related death was defined as diagnosis of dying from AIDS-related tumors, AIDS-related opportunistic infections, AIDS-related syndrome, or other AIDS-related diseases. All the other causes of death were classified as non-AIDS-related deaths. Patients alive until May 30, 2016, or those lost during the follow-up period, were defined as censored data. Survival time was calculated from the date of HIV diagnosis to the date of death.

### Statistical analysis

Cumulative incidence function (CIF) was used to estimate the probability of AIDS-related mortality [8]. The sub-distribution proportional hazard model of Fine and Gray [11] was applied to assess the effects of covariates on the CIF for AIDS-related deaths. Significant variables in the univariate analysis were included in the multivariate competing risk model. The proportional hazards (PH) assumption was verified based on weighted Schoenfeld residual analysis.

The model’s discrimination was assessed by the time-dependent receiver operating characteristic (time-dependent ROC) curve. The area under the time-dependent ROC curve (AUC(t)) of more than 0.75 clearly reflected useful discrimination, 0.60 to 0.75 reflected possibly helpful discrimination, and less than 0.60 reflected poor discrimination [12].

All statistical analyses were performed using R version 3.5.2 (R Foundation for Statistical Computing, Vienna, Austria, https://www.R-project.org/). The significance level was *α* = 0.05.

## Results

### Characteristics of HIV/AIDS patients

11,905 HIV/AIDS patients were followed up for 89,070 person-years. The median follow-up time was 8.93 years (interquartile range [IQR] 3.60–11.33 years). In the whole cohort, 3,198 (26.86%) died of AIDS-related diseases, 607 (5.10%) died owing to non-AIDS-related causes, while 8,100 (68.04%) were still alive or lost to follow-up. 7,220 (60.65%) patients had already progressed to AIDS at the time of diagnosis (Table 1).

**Table 1.** Characteristics of HIV/AIDS patients [n(%)]

Variable	Total	Censored	AIDS related mortality	Non-AIDS related mortality
Gender				
Female	5713(47.99)	4122(50.89)	1363(42.62)	228(37.56)
Male	6192(52.01)	3978(49.11)	1835(57.38)	379(62.44)
Age at diagnosis (years)				
15–29	1056(8.87)	814(10.05)	212(6.63)	30(4.94)
30–44	6685(56.15)	4862(60.02)	1558(48.72)	265(43.66)
45–59	3474(29.18)	2091(25.82)	1168(36.52)	215(35.42)
≥60	690(5.80)	333(4.11)	260(8.13)	97(15.98)
Educational level				
Illiteracy	1431(12.02)	787(9.72)	565(17.67)	79(13.01)
Primary school	5537(46.51)	3739(46.16)	1546(48.34)	252(41.52)
Junior high school	4501(37.81)	3266(40.32)	994(31.08)	241(39.70)
Senior high school and above	436(3.66)	308(3.80)	93(2.91)	35(5.77)
Marital status				
Single	747(6.27)	430(5.31)	272(8.51)	45(7.41)
Married	8500(71.40)	5797(71.57)	2319(72.51)	384(63.26)
Divorced or widowed	2658(22.33)	1873(23.12)	607(18.98)	178(29.33)
Occupation				
Farmer	11309(94.99)	7669(94.68)	3080(96.31)	560(92.26)
Others	596(5.01)	431(5.32)	118(3.69)	47(7.74)
Transmission				
Sexual	2150(18.06)	1616(19.95)	380(11.88)	154(25.37)
Blood	9575(80.43)	6376(78.72)	2756(86.18)	443(72.98)
Others	180(1.51)	108(1.33)	62(1.94)	10(1.65)
Disease stage at diagnosis				
HIV	4685(39.35)	3734(46.10)	754(23.58)	197(32.45)
AIDS	7220(60.65)	4366(53.90)	2444(76.42)	410(67.55)
Time from HIV diagnosis to ART initiation				
Immediate ART (≤90 days)	7713(64.79)	5633(69.54)	1737(54.32)	343(56.51)
Delayed ART (>90 days)	2808(23.59)	2113(26.09)	589(18.42)	106(17.46)
Late ART (>1 year)	1384(11.63)	354(4.37)	872(27.27)	158(26.03)
Baseline CD4^+^ cell counts (cells/μL)				
≤200	5282(44.37)	3169(39.12)	1821(56.94)	292(48.10)
201–350	2876(24.16)	2280(28.15)	469(14.66)	127(20.92)
351–500	1548(13.00)	1277(15.77)	202(6.32)	69(11.37)
>500	1005(8.44)	867(10.70)	101(3.16)	37(6.10)
Untested	1194(10.03)	507(6.26)	605(18.92)	82(13.51)

ART: antiretroviral therapy; HIV: human immunodeficiency virus; AIDS: acquired immune deficiency syndrome.

### Cumulative incidence in groups with different characteristics

**Figure 1.**
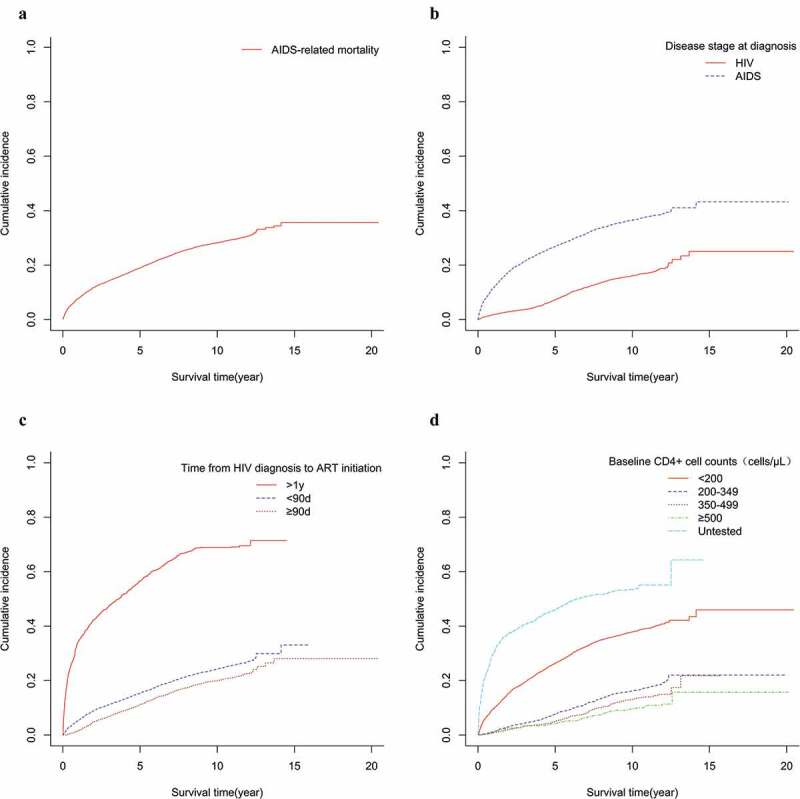
Cumulative incidence curves of AIDS-related mortality in Henan province: 1995–2016 (*N* = 11,905). ((a) Cumulative incidence curves of AIDS-related mortality; (b) Cumulative incidence curves of AIDS-related mortality for HIV patients and AIDS patients; (c) Cumulative incidence curves of AIDS-related mortality for patients with initiation of ART and ART-naive; (d) Cumulative incidence curves of AIDS-related mortality for patients with different CD4^+^ cell counts)

ART: antiretroviral therapy; HIV: human immunodeficiency virus; AIDS: acquired immune deficiency syndrome.

Cumulative incidence of AIDS-related death at one, two, five, ten, and 15 years after diagnosis was 9.8%, 12.9%, 19.3%, 26.0%, and 26.9%, respectively (Figure 1(a)). Patients diagnosed with AIDS had a higher cumulative incidence (Figure 1(b)). Cumulative incidence of AIDS-related death in ART initiation time >one year was the highest (Figure 1(c)). Patients with higher baseline CD4^+^ cell counts had a lower cumulative incidence of AIDS-related death (Figure 1(d)).

### AIDS-related mortality by categories of ART initiation time and baseline CD4^+^ cell counts

**Figure 2.**
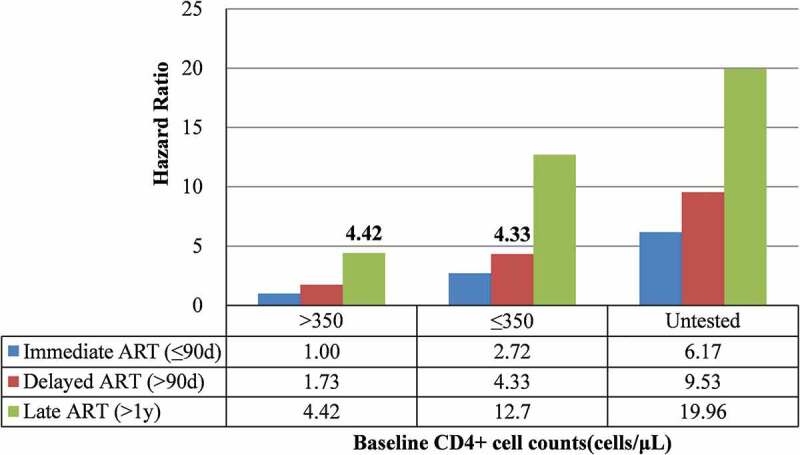
AIDS-related mortality stratified by time of ART initiation and baseline CD4^+^ cell counts. (Competing risk model adjusted for gender, age, educational level, marital status, occupation, transmission, and disease stage at diagnosis)

ART: antiretroviral therapy; AIDS: acquired immune deficiency syndrome.

Results of stratified analysis according to baseline CD4^+^ cell counts were presented in Table 2. There were 79% and 68% decreases in AIDS-related mortality risk in immediate ART and delayed ART compared with late ART in both high and low CD4^+^ cell count groups. Being male, older, having a low educational level, and not being married were risk factors for AIDS-related mortality in both high and low CD4^+^ cell count groups. Figure 2 showed AIDS-related mortality by categories of ART initiation time and baseline CD4^+^ cell counts, adjusted for confounding factors. Significantly higher sub-distribution hazard ratios (*sHR*s) were found with an increase of ART time and a decrease in CD4^+^ cell counts. ART initiation time >one year and CD4^+^ cell counts >350 (*sHR*: 4.42, 95% *CI*: 3.30–5.91) presented higher AIDS-related mortality risks than ART initiation time of >90 days and CD4^+^ cell counts ≤350 (*sHR*: 4.33, 95% *CI*: 3.58–5.23).

**Table 2.** Prognostic factors of entire study cohort and each subgroup based on baseline CD4^+^ cell counts [*sHR* (95% *CI*)]

		Baseline CD4^+^ cell counts (cells/μL)	
Variable	Entire study cohort	Low (≤350)	High (>350)
Gender			
Female	1(Reference)	1(Reference)	1(Reference)
Male	1.32(1.22–1.43)	1.34(1.11–1.63)	1.45(1.13–1.85)
Age at diagnosis (years)			
15–29	1(Reference)	1(Reference)	1(Reference)
30–44	1.14(0.98–1.33)	1.56(0.97–2.52)	1.31(0.80–2.14)
45–59	1.78(1.52–2.10)	3.44(2.11–5.61)	2.49(1.48–4.18)
≥60	1.97(1.59–2.46)	6.68(3.63–12.29)	9.04(4.75–17.22)
Educational level			
Illiteracy	1(Reference)	1(Reference)	1(Reference)
Primary school	0.76(0.68–0.85)	0.82(0.63–1.06)	0.70(0.50–0.96)
Junior high school	0.69(0.61–0.78)	0.81(0.60–1.08)	0.61(0.42–0.88)
Senior high school and above	0.68(0.53–0.88)	0.79(0.39–1.58)	0.52(0.20–1.32)
Marital status			
Single	1(Reference)	1(Reference)	1(Reference)
Married	0.82(0.69–0.97)	0.82(0.63–1.06)	0.45(0.29–0.70)
Divorced or widowed	0.56(0.47–0.68)	0.81(0.60–1.08)	0.42(0.26–0.68)
Occupation			
Others	1(Reference)	1(Reference)	1(Reference)
Farmer	1.32(1.05–1.66)	1.56(0.76–3.23)	1.42(0.59–3.41)
Transmission			
Sexual	1(Reference)	1(Reference)	1(Reference)
Blood	1.57(1.37–1.80)	1.19(0.83–1.71)	1.14(0.75–1.73)
Others	1.34(0.98–1.83)	2.13(0.64–7.15)	1.17(0.22–6.30)
Disease stage at diagnosis			
HIV	1(Reference)	1(Reference)	1(Reference)
AIDS	2.48(2.23–2.75)	2.28(1.77–2.93)	2.05(1.49–2.83)
Time from HIV diagnosis to ART initiation			
Late ART (>1 year)	1(Reference)	1(Reference)	1(Reference)
Immediate ART (≤90d)	0.24(0.22,0.27)	0.21(0.15–0.28)	0.21(0.15–0.28)
Delayed ART (>90d)	0.38(0.33,0.43)	0.32(0.22–0.45)	0.32(0.21–0.47)
Baseline CD4^+^ cell counts(cells/μL)			
≤200	1(Reference)		
201–350	0.43(0.39–0.48)		
351–500	0.34(0.29–0.39)		
>500	0.23(0.19–0.28)		
Untested	1.57(1.39–1.77)		

ART: antiretroviral therapy; HIV: human immunodeficiency virus; AIDS: acquired immunodeficiency syndrome; *sHR*: sub-distribution hazard ratio; *CI*: confidence interval.

### Predictors of AIDS-related mortality

The results of the entire study cohort using the competing risk regression model were shown in Table 2. Earlier ART provided stronger protection against death (*sHR*: 0.24, 95% *CI*: 0.22–0.27), while delayed ART provided more protection (*sHR*: 0.38, 95% *CI*: 0.33–0.43) than late ART. Higher baseline CD4^+^ cell counts were associated with lower AIDS-related mortality. In addition, being male (*sHR*: 1.32, 95% *CI*: 1.22–1.43), older (*sHR*: 1.97, 95% *CI*: 1.59–2.46), being a farmer (*sHR*: 1.32, 95% *CI*: 1.05–1.66), AIDS stage at diagnosis (*sHR*: 2.48, 95% *CI*: 2.23–2.75), and infection by blood transmission (*sHR*: 1.57, 95% *CI*: 1.37–1.80) were all associated with an increased risk of AIDS-related death. However, high educational level and marriage were protective factors for AIDS-related death.

### Model performance

**Figure 3.**
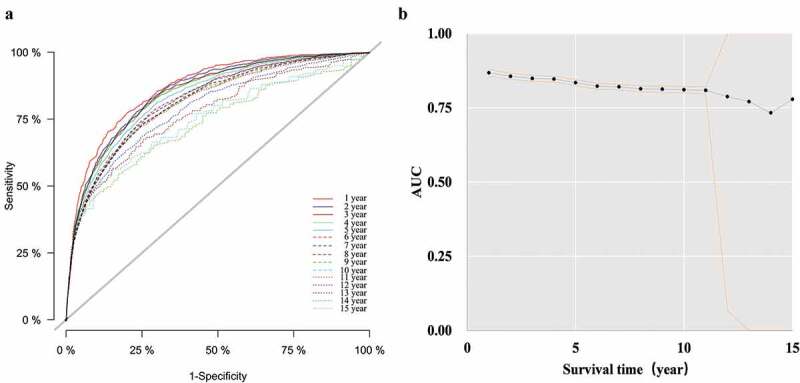
Model performance of entire study cohort based on time-dependent ROC. ((a) time-dependent ROC curve; (b) AUC(t) values and 95% *CI*)

As shown in Figure 3, AUC(t) values were above 0.73 in the 15 years after diagnosis, and the overall model performance was good.

Time-dependent ROC: time-dependent receiver operating characteristic curve; AUC(t): the area under time-dependent receiver operating characteristic curve; *CI*: confidence interval.

## Discussion

In the present study, results showed that there were 79% and 68% decrease in AIDS-related mortality risk with immediate ART and delayed ART, respectively, compared with late ART, whether in patients with high CD4^+^ cell counts or low counts. Patients with CD4^+^ cell counts >350 who initiated ART after one year had 9% higher risk of AIDS-related death than those with CD4^+^ cell counts ≤350 whose ART initiation time was >90 days. Additional risk factors for mortality in this study were being male, being of older age, being a farmer, the patient’s AIDS stage at diagnosis, having been infected by blood transmission, having a low level of education, and being single. Our results were consistent with the WHO’s guidelines on the use of antiretroviral drugs for treating and preventing HIV infection.

The mechanism by which ART decreased the mortality risk was primarily through viral suppression and improved immunological recovery [13]. A strategy of earlier initiation of ART based on CD4^+^ cell counts has been reported as preventing HIV transmission and reducing the rate of clinical events [14]. CD4^+^ cell count is the most important laboratory indicator of immune function. When CD4^+^ cell counts decline below a certain level, HIV-infected individuals are at risk of immune deficiency and more susceptible to infection, resulting in progress to AIDS or death [15]. This study indicated risk of AIDS-related mortality decreased with an earlier ART initiation time after controlling for CD4^+^ cell counts and other confounders. Patients with high baseline CD4^+^ cell counts who initiated ART after one year had 9% higher risk of AIDS-related death than those with lower baseline CD4^+^ cell counts who initiated ART after 90 days but within one year of diagnosis. Patients with higher CD4^+^ levels at baseline who missed treatment opportunities had a worse prognosis than those with lower CD4^+^ levels at baseline [16,17]. Arguments for delaying ART initiation based on the use of a CD4^+^ cell count threshold include concerns about drug resistance, side effects, and resource allocation [18–23]. However, previous studies have concluded that the benefits of immediate ART initiation outweigh its disadvantages. Zhao et al. [24] discovered that immediate ART when CD4^+^ counts were >500 cells/μL could reduce the one-year mortality. Our results emphasized the importance of immediate ART initiation for long-term survival of former plasma donors with high and low CD4^+^ cell counts. Early screening and immediate treatment after diagnosis should therefore be advocated, including voluntary testing, counselling, and entry into the Chinese National Free Antiretroviral Treatment Program [25].

Illegal commercial plasma and blood collection activities were very common in rural central China before the national blood donation law was enacted [26]. In the present study, 76.0% of HIV/AIDS patients among FPD were infected through blood transmission. Patients infected via blood transmission had higher plasma HIV loads and lower CD4^+^ cell counts than patients infected by sexual transmission, resulting in faster progression of AIDS or earlier death [4,27]. Higher mortality among older males may be attributed to their late diagnosis, comorbidities, and poor immunological responses to ART [28,29]. Educational level was negatively associated with AIDS-related mortality, probably because individuals with high education were more likely to adopt protective measures than others [30,31]. Lower AIDS-related mortality risk was observed among married patients, which related with fewer sexual partners [32].

Discrimination is an important characteristic in the evaluation of model performance [33]. In the presence of competing events, discrimination is typically characterized using the time-dependent ROC curve [12,34]. In this study, the AUC(t) values of each year above 0.80 were in the 11 years after diagnosis according to the multivariate competing risk model. After 12 years of follow-up, the AUC(t) values decreased, and the 95%*CI* expanded. A possible reason is that the new reported mortality numbers after 12 years were very low (the numbers of AIDS-related death at 12, 13, 14, and 15 years after diagnosis were 54, eight, two, and zero, respectively). Under these circumstances, the competitive risk model is inapplicable in evaluating the prognostic factors of AIDS-related mortality.

The present study has several important strengths. Firstly, as far as we know, it is the first study to assess competing risk performance using the time-dependent ROC curve in AIDS-related mortality studies. It could avoid overestimation of cumulative incidence and bias in the effects of covariates. Secondly, our study had a large sample size (n = 11,905) and long follow-up time (21 years). Lastly, we explored the association between time from HIV diagnosis to ART initiation and AIDS-related mortality in HIV/AIDS patients with high and low CD4^+^ cell counts. Inevitably, this study has some limitations. We have not considered dynamic changes in CD4^+^ cell counts during follow-up, which may influence survival in HIV/AIDS patients. In addition, compliance with ART, which may influence HIV/AIDS prognosis, was not included in the present analysis because of unavailability of data. In future studies, details of the dynamic changes in CD4^+^ cell counts and ART compliance should be considered in order to explore their influence on AIDS-related mortality.

## Conclusion

Our results demonstrate the urgent need to advocate early ART for improving survival of HIV/AIDS patients, whether with high or low CD4^+^ cell counts, especially for elders, males, and patients infected through blood transmission. A competing risk model performs well in the short and medium term, providing a reference method for the development of HIV/AIDS risk prediction models in the future.

## Paper context

The World Health Organization recommends lifelong antiretroviral therapy for all HIV/AIDS patients to reduce AIDS-related mortality. In this study, the competing risk model was used to explore the association between time from HIV diagnosis to antiretroviral therapy initiation and AIDS-related mortality in HIV/AIDS patients with high and low CD4^+^ cell counts. This model can provide a reference method for the construction of HIV/AIDS risk prediction models in the future.

## Preprint

The effect of antiretroviral therapy initiation time and baseline CD4+ cell counts on AIDS-related mortality among former plasma donors in China: a 20-Year retrospective cohort study, also known as the Author’s Original Manuscript (AOM) is available online https://www.researchsquare.com/article/rs-13919/v1 [Article DOI:10.1080/16549716.2021.1963527].
